# Whole-exome sequencing identified recurrent and novel variants in benzene-induced leukemia

**DOI:** 10.1186/s12920-023-01442-w

**Published:** 2023-01-26

**Authors:** Dafeng Lin, Dianpeng Wang, Peimao Li, Lihua Deng, Zhimin Zhang, Yanfang Zhang, Ming Zhang, Naixing Zhang

**Affiliations:** 1 Occupational Health Department, Shenzhen Prevention and Treatment Center for Occupational Diseases, 2019 Buxin Rd., Luohu District, Shenzhen, 518020 China; 2 Medical Laboratory, Shenzhen Prevention and Treatment Center for Occupational Diseases, Shenzhen, 518020 China; 3 Occupational Diseases Department, Shenzhen Prevention and Treatment Center for Occupational Diseases, Shenzhen, 518020 China

**Keywords:** Benzene, Exome, High-throughput sequencing, Leukemia, Genetic variant

## Abstract

**Background:**

Genome-wide sequencing may extensively identify potential pathogenic variants, which helps to understand mechanisms of tumorigenesis, but such study has not been reported in benzene-induced leukemia (BIL).

**Methods:**

We recruited 10 BIL patients and conducted the whole-exome sequencing on their peripheral blood samples. The obtained sequencing data were screened for potential pathogenic and novel variants, then the variants-located genes were clustered to identify cancer-related pathways. Shared or recurrent variants among the BIL cases were also identified and evaluated for their potential functional impact.

**Results:**

We identified 48,802 variants in exons in total, 97.3% of which were single nucleotide variants. After filtering out variants with minor allele frequency ≥ 1%, we obtained 8667 potentially pathogenic variants, of which 174 were shared by all the BIL cases. The identified variants located in genes that could be significantly enriched into certain cancer-related pathways such as PI3K-AKT signaling pathway and Ras signaling pathway. We also identified 1010 novel variants with no record in the Genome Aggregation Database and in dbSNP, and one of them was shared by 90% cases. The recurrent and novel variant caused a missense mutation in *SESN3*.

**Conclusions:**

We examined variations of the whole exome in BIL patients for the first time. The commonly shared variants implied a relation with BIL, and the recurrent and novel variant might be specifically related to BIL. The related variants may help unravel the carcinogenic mechanisms of BIL.

**Supplementary Information:**

The online version contains supplementary material available at 10.1186/s12920-023-01442-w.

## Background

Benzene has been wildly used as chemical intermediate and organic solvent in the chemical, petroleum and drug industries all over the world for about a century, though it was gradually proved to have various adverse health effects, especially to the haematopoietic system [1]. Carcinogenicity of benzene was first established in 1982 based on definite evidence of animal experiments as well as epidemiological studies [1], and benzene-induced leukemia (BIL) is diagnosed in China according to the current national criteria GBZ 94-2017 “Diagnosis of Occupational Tumor” [2]. Apart from exposure to benzene of over 6 mg/m^3^ for at least 6 months, the diagnosis requires a latent period of 2 or more years [2]. Besides, leukemia developed with a medical history of chronic benzene poisoning can be directly diagnosed as BIL [2]. Large cohort studies estimated that the incidence of leukemia among benzene exposure workers was 13.59 per 100,000, and the relative risk for all hematologic neoplasms combined was 2.6 (95% CI 1.4–4.7) [3, 4].

It was reported that about 80% of BIL were clinically classified as acute myeloid leukemia (AML), and about 20% were acute lymphocytic leukemia (ALL) and chronic myeloid leukemia (CML) combined [5, 6]. We observed more CML in clinics than ALL. Both AML and CML are malignant bone marrow cancers showing the characteristic of abnormal growth of myeloid cells at different maturation stages. Clinically, CML can be classified into chronic phase (CP), accelerated phase (AP), and blast phase (BP) or blast crisis (BC) [7]. Although most CML patients remain in the CP which is relatively benign, their conditions will undesirably progress to the myeloid or lymphoid BP if left untreated. CML in BP is similar to AML, both of them show an increased frequency of blast cells, aggressiveness and poor prognosis [8]. The Philadelphia chromosome (Ph) is a unique hallmark of CML cells, which results from the t(9;22)(q34;q11) chromosomal translocation and encodes a carcinogenic fusion protein BCR-ABL1, a tyrosine kinase with deregulated activity. The tyrosine kinase inhibitors (TKIs) targeting BCR-ABL1 were developed 2 decades ago to effectively treat CML [8]. Comparatively, AML was found to have at least 24 different genetic subtypes, suggesting quite more heterogeneous molecular features than CML [9, 10]. Until this day, cytotoxic chemotherapy is still the same standard treatment for AML as in half a century ago [8].

The key to develop more efficient cancer detection methods and therapeutic approaches is uncovering the mechanisms of tumorigenesis, which is considered primarily driven by genetic mutations [11]. Nowadays genetic studies have reached to the genome-wide scope at a single-base resolution thanks to the next-generation sequencing technologies. AML was among the first malignancies that had been extensively studied by novel high-throughput microarray and sequencing technologies [12]. After first reported in 2008, genomic studies in AML identified numerous novel recurrent somatic alleles, such as mutations in the DNA methyltransferase 3A gene (*DNMT3A*) and the isocitrate dehydrogenase genes (*IDH1* and *IDH2*), and most of them were of biologic, prognostic, or therapeutic relevance [12]. As for CML, studies focused on detecting mutations in *BCR-ABL1* gene, because the fusion gene plays an important role in the pathogenesis of CML, and mutations in it might engender failure of one or more of the currently effective TKIs [13]. Such reported mutations include T315I, Y253F/H, E255K, V299L and L237M, etc. (transcript ENST00000318560.6). [13]. Besides, studies also discovered mutations in *ASXL1*, *DNMT3A*, *EZH2*, *RUNX1*, *TET2* and *TP53* genes in the chronic phase of CML, and in *CBL*, *CDKN2A*, *GATA-2*, *IDH1*, *IDH2*, *IKZF1*, *KRAS*, *NPM1*, *NRAS*, *RB1*, *RUNX1*, *TET2*, *TP53* and *WT1* genes in the advanced phase [13, 14]. Unfortunately, there has been no genome-wide sequencing studies reported on BIL to date regardless of the clinical types.

While being clinically heterogeneous, BIL has the same oncogenous origin, that is benzene exposure. Whether there are shared (or recurrent) genetic variants among the BIL patients is still unknown to the best of our knowledge. Furthermore, if the putative variants were specific to BIL and/or had potential impact on biological structures or functions, they would be quite valuable for further studies on carcinogenic mechanisms. Intrigued by the aforementioned hypothesis, we tentatively recruited 10 BIL patients and conducted the whole-exome sequencing (WES) on their peripheral blood samples. The sequencing data were first screened for potential pathogenic variants by using a cutoff value of minor allele frequency (MAF) < 1%, and then screened for novel variants by excluding those recorded in the Genome Aggregation Database (gnomAD) and in Database of Short Genetic Variation (dbSNP). The pathogenic variants- and the novel variants-located genes were separately evaluated using the Kyoto Encyclopedia of Genes and Genomes (KEGG) database to identify cancer related pathways. Thereafter, commonly shared pathogenic variants and recurrent novel variants among the BIL cases were preliminarily examined for their potential functional impact.

## Methods

### Cases information and samples

We recruited 10 BIL cases from all 39 leukemia patients hospitalized in Shenzhen Prevention and Treatment Center for Occupational Diseases (SPTCOD) during the year 2016–2020, excluded were non-BIL and patients undergone hematopoietic stem cell transplantation therapy. BIL was strictly diagnosed according to the GBZ 94-2017 criteria [2]. Originally, we planned to select 5 CML and 5 AML from the BIL cases successively by date of admission, but only 4 AML cases met the selection criteria, so we finally included one more CML to get 10 study cases in total. Written informed consent for participating the study was obtained from all the cases or their guardians.

We collected and sorted demographic, occupational and medical information of the cases from their electronic medical records. Peripheral blood samples of the cases were collected after their routine laboratory examination during the hospitalization, and stored at − 80 °C for later use. Unfortunately, paired normal samples were not obtained at the same time. The use of patient information and peripheral blood samples for further studies beyond routine laboratory examination was approved by the Ethics Committee of SPTCOD (No. LL-202036). This study abides by the Helsinki Declaration on ethical principles for medical research involving human subjects.

### Whole-exome sequencing

Extracted from 1.0 mL of each blood sample, each genomic DNA sample was quantified by using a spectrofluorometer (Gemini™ XPS Microplate Reader, Molecular Devices, USA), and about 200 ng of each DNA sample were randomly fragmented by Covaris sonication (LE220-plus Focused-ultrasonicator, Covaris, USA). The DNA fragments with the size mainly distributed between 150 and 250 bp were repaired with an "A" base added at the 3′-end of each strand, thereafter adapters were ligated to both ends of the end repaired/dA tailed DNA fragments, which were selected by size, amplified by ligation-mediated PCR (S1000 Thermal Cycler, Bio-Rad, USA), purified [QIAquick PCR Purification Kit, QIAGEN China (Shanghai)], and hybridized to the exome array for enrichment. After washing out non-hybridized fragments, captured products were circularized and the rolling circle amplification was performed to produce DNA nanoballs. Each resulting qualified captured library was then loaded on BGISEQ-500 sequencing platforms (MGI Tech, China) to perform high-throughput sequencing [15, 16]. Sequencing-derived raw image files were processed by BGISEQ-500 base calling software with default parameters to generate the sequence data of each case as paired-end reads.

### Data analysis and plotting

The raw sequencing data were first filtered to obtain high-quality clean data by the following methods: (a) removing the adapter sequences from all reads, (b) removing the pair of reads if the percentage of low-quality base in either of the two end reads exceeds 50% or the percentage of N base in either of the two end reads exceeds 10%. All clean data of each sample were then mapped to the human reference genome (GRCh38) using Burrows–Wheeler Aligner (BWA V0.7.15) [17, 18]. Local realignment around small insertions and deletions (InDels) and base quality score recalibration were performed using the Genome Analysis Toolkit (GATK) [19, 20], with duplicate reads removed by Picard tools [21]. The strict data analysis quality control system was built through the whole process to guarantee qualified sequencing data. All genomic variations including single nucleotide variants (SNVs) and InDels were detected by the state-of-the-art software, such as HaplotypeCaller of GATK (v3.7). After that, the hard-filtering method was applied to get high-confident variant calls, and the SnpEff tool was applied to perform a series of annotations for variants [22, 23]. Detailed description of the bioinformatics analysis has been reported before [15, 16].

Advanced data analysis and plotting were performed using R v4.0.4 [24]. We first used MAF < 1% as a cutoff value to filter out potential polymorphic or benign variants, and the obtained variants were examined for their located genes, which were then put into KOBAS v3.0 for pathway enrichment using the KEGG database with *P* value and corrected *P* value both < 0.05 [25]. Commonly shared variants among the BIL cases were identified and assessed for their functional impact. Subsequently, we further excluded those recorded in dbSNP and in gnomAD to get novel variants, and the variants-located genes were subjected to a similar pathway analysis. Thereafter, the recurrent novel variants were examined for their potential impact on normal functions of the genes.

## Results

### Basic information of the cases

The 10 BIL cases had an average age of 44 years old, and 30% of them were male. Only one male patient (10%) had smoking and drinking habits. The mean time of benzene exposure of the cases was 6.47 years, and showed no significant difference between the AML and BML cases (*P* = 0.459). The *BCR-ABL1* gene had been detected in all the 6 CML cases before their TKIs therapy, but the fusion gene was not detected in the AML cases who were therefore treated with the chemotherapy. The cases were all alive at the end of follow-up, and the average of survival time (from date of clinical diagnosis to end of follow-up) was 8.56 years for all cases, and was not significantly different between the AML and BML cases (*P* = 0.173). The blood cell indices of the samples used for WES, including counts of white blood cell, neutrophil and platelet, and immature granulocyte percentage were all within their clinical references, respectively; the indices were not significantly different between the AML and BML cases (*P* = 0.091, 0.156, 0.591 and 0.507, respectively). Detailed information of the 10 BIL cases was listed in Table 1.

**Table 1:** Basic information of the benzene-induced leukemia cases

Variables	Total (*n* = 10)	Clinical diagnosis	
		AML (*n* = 4)	CML (*n* = 6)
Age [year, mean (SE)]	44.00 (1.69)	44.25 (3.01)	43.83 (2.21)
Gender [male (%)]	30.00	25.00	33.33
Race [Han (%)]	100.00	100.00	100.00
Smoking [yes (%)]	10.00	25.00	0
Drinking [yes (%)]	10.00	25.00	0
Benzene exposure duration [year, mean (SE)]	6.47 (1.13)	7.93 (1.92)	5.74 (1.46)
White blood cell count [× 10^9^/L, mean (SE)]	4.77 (0.68)	6.50 (1.20)	3.62 (0.41)
Neutrophil count [× 10^9^/L, mean (SE)]	2.76 (0.53)	3.95 (1.05)	1.97 (0.28)
Platelet count [× 10^9^/L, mean (SE)]	187.90 (24.95)	208.25 (52.69)	174.33 (25.79)
Immature granulocyte percentage [%, mean (SE)]	0.17 (0.05)	0.12 (0.08)	0.20 (0.08)
*BCR-ABL1* fusion gene [positive (%)]	60.00	0	100.00
Treatment			
Chemotherapy (%)	40.00	100.00	0
TKIs therapy (%)	60.00	0	100.00
Survival time [year, mean (SE)]	8.56 (0.66)	7.52 (0.62)	9.24 (0.96)

*AML* acute myeloid leukemia, *CML* chronic myeloid leukemia, *SE* standard error

### Overview of the WES-identified variants

The WES yielded an average of 138,116,902 raw reads per sample. After removing low-quality reads, we obtained averagely 137,989,040 clean reads per sample, thus the mean clean data rate was 99.9%. The clean reads of each sample had high Q20 and Q30, showing high sequencing quality. The average GC content was 49.64%. All WES data production was summarized in Additional file 1.

On average, we captured 60.46 Mb target region, and successfully mapped 99.93% of the clean reads to the human reference genome. After removing the duplicate reads, we obtained a mean of 114,812,614 effective reads. The capture specificity that is the percentage of total effective bases mapped on target regions was 50.55%. We attained a 95.49 fold of mean sequencing depth on target regions. On average, 99.42% of targeted bases per sample were at least sequenced by 1 × coverage and 98.73% by 10 × coverage (see Additional file 2).

As shown in Table 2, we identified averagely 20,213 InDels and 121,407 SNVs per sample. The mean numbers of novel InDels and SNVs presented neither in dbSNP nor in gnomAD were 354 and 195, respectively. Of the InDels on average, 250 were frameshift, 82 were non-frameshift insertion, 120 were non-frameshift deletion, 2 were startloss, and 53 were splice site; of the SNVs on average, 10,467 were synonymous, 10,397 were missense, 40 were stoploss, 113 were stopgain, 33 were startloss, and 153 were splice site.

**Table 2:** Statistics of the whole-exome sequencing identified variants

Variant type	Sample	Total variants	Fraction in dbSNP (%)	Fraction in gnomAD (%)	Novel	Homozygous	Heterozygous	Intronic	5′-UTR	3′-UTR	Upstream	Downstream	Intergenic
InDel	Case_1	20,008	94.59	87.02	332	7535	12,473	15,959	307	750	752	568	149
	Case_2	20,168	94.98	86.47	324	7666	12,502	16,072	333	761	792	558	138
	Case_3	19,731	95.61	87.55	261	7784	11,947	15,823	311	715	738	581	129
	Case_4	20,758	95.10	86.65	324	7803	12,955	16,624	336	770	809	566	132
	Case_5	20,193	95.40	85.43	327	7915	12,278	16,149	338	769	782	557	113
	Case_6	20,220	95.51	81.64	397	7781	12,439	16,188	330	748	762	564	126
	Case_7	20,149	94.95	83.30	405	7596	12,553	16,167	317	750	735	548	133
	Case_8	19,991	95.19	84.22	364	7904	12,087	16,060	324	720	777	556	122
	Case_9	20,822	94.60	81.88	477	7596	13,226	16,712	333	757	804	579	125
	Case_10	20,095	94.79	86.69	329	7601	12,494	16,129	319	720	737	548	120
SNV	Case_1	120,593	99.62	97.73	165	52,586	68,007	82,056	2249	3689	4236	2808	475
	Case_2	120,805	99.60	97.59	198	52,520	68,285	82,454	2309	3729	4280	2900	431
	Case_3	121,785	99.62	97.66	187	53,641	68,144	82,746	2308	3780	4315	2860	458
	Case_4	123,072	99.62	97.87	160	52,059	71,013	84,168	2330	3805	4313	2902	444
	Case_5	122,073	99.61	97.68	178	52,677	69,396	83,687	2325	3797	4238	2807	418
	Case_6	121,331	99.62	97.57	201	52,564	68,767	83,004	2323	3768	4269	2797	439
	Case_7	120,998	99.60	97.88	168	51,671	69,327	82,535	2310	3752	4183	2795	455
	Case_8	120,987	99.60	97.71	182	52,956	68,031	82,752	2285	3647	4210	2890	461
	Case_9	121,267	99.58	97.61	201	52,311	68,956	83,191	2265	3709	4257	2828	457
	Case_10	121,166	99.35	97.48	309	52,751	68,415	82,685	2327	3720	4174	2790	450

*dbSNP* Database of Short Genetic Variation, *gnomAD* genome aggregation database, *InDel* small insertion and deletion, *SNV* single nucleotide variant

### Variants with minor allele frequency < 1%

To filter out potential polymorphic or benign variants in the absence of paired normal samples, we used MAF < 1% as a cutoff value, which is recommended by the Interpretation of Sequence Variants in Somatic Conditions Working Group and also commonly used across many clinical laboratories [26]. In total, we identified 8667 variants with MAF < 1% or not recorded in gnomAD for East Asian. Ninety-three percent of them were SNVs, and 14.8% and 4.5% were recorded in Catalogue of Somatic Mutations in Cancer (COSMIC) and ClinVar databases, respectively. Those variants were listed in Additional file 3.

**Fig. 1:**
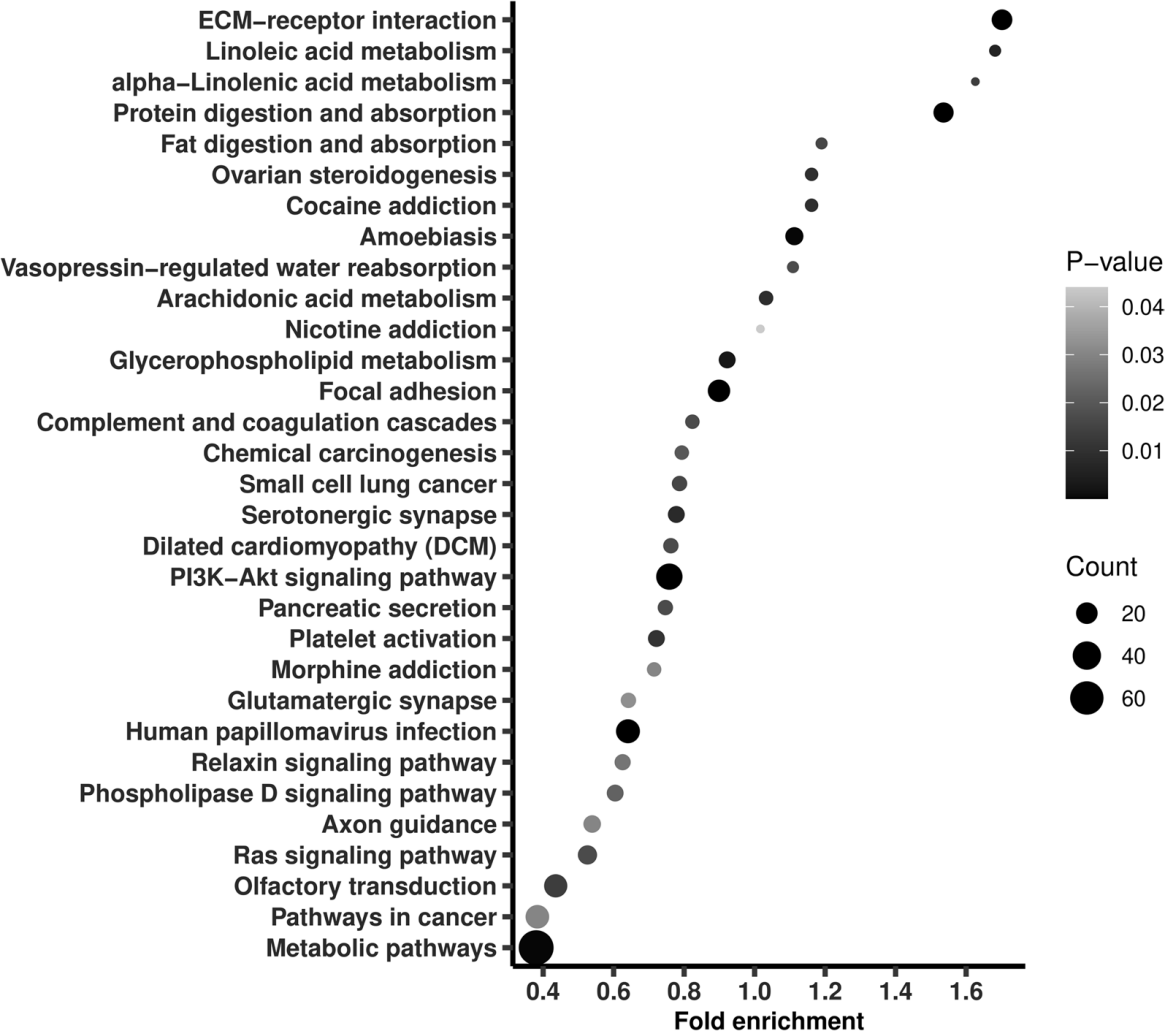
Pathway enrichment of the genes where the identified variants with minor allele frequency < 1% located. Fold enrichment is calculated by dividing gene ratio (the number of genes enriched in the pathway divided by the total number of analyzed genes) with background ratio in KEGG database. *P* values were adjusted

The 8667 variants located in 5665 genes, of which *PABPC3* had the largest number of variants, that was 48. Of all the genes, 998 were recorded in COSMIC database, on which we thereafter conducted a pathway enrichment. As shown in Fig. 1, the genes were enriched into 31 pathways, including metabolic pathways, pathways in cancer, PI3K-AKT signaling pathway, and Ras signaling pathway. Notably, metabolic pathways had the largest gene ratio (0.067), and PI3K-AKT signaling pathway had the smallest adjusted *P* value (9.78 × 10^−8^).

**Fig. 2:**
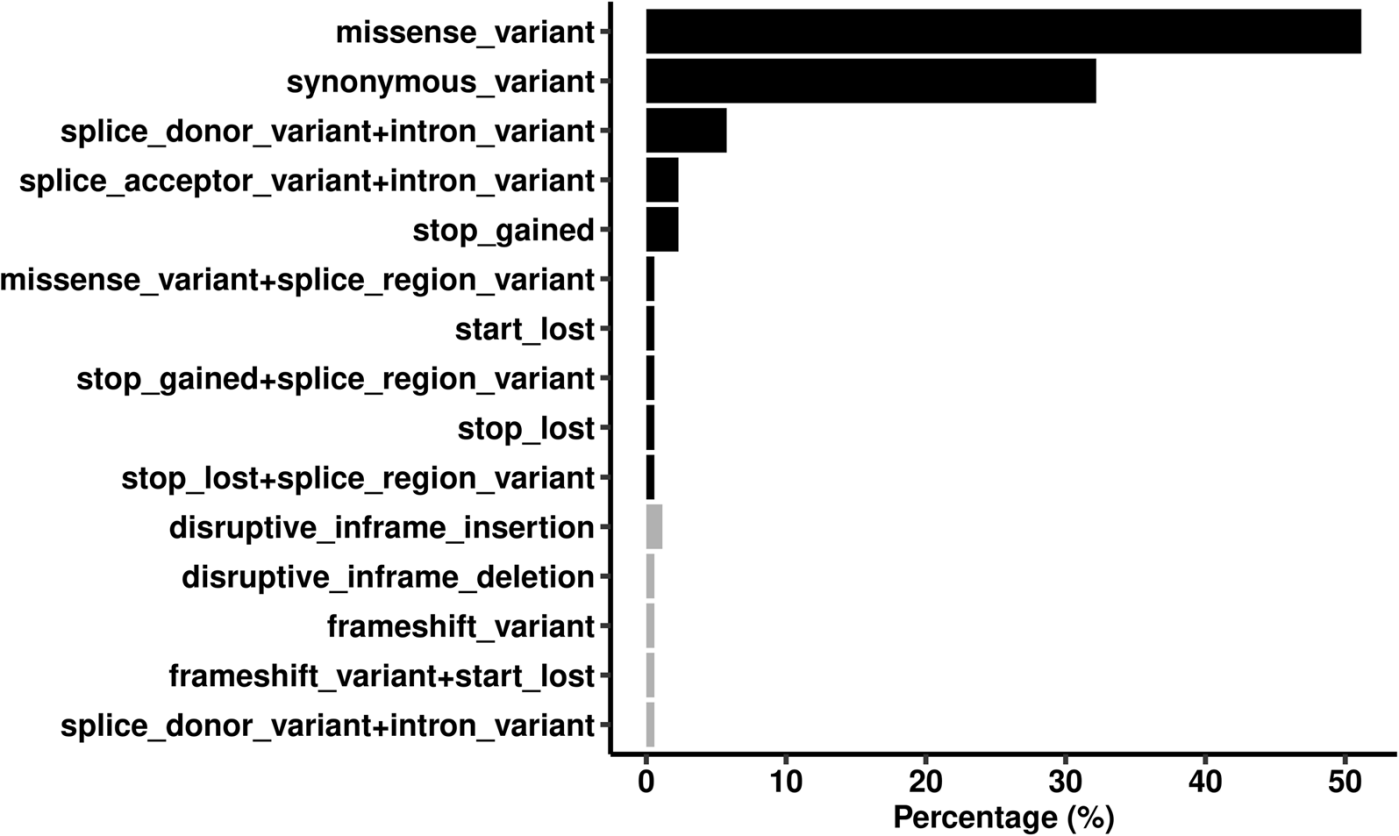
Classification of the functional impact of the commonly shared variants with minor allele frequency < 1%. Black bars represent single nucleotide variants, and gray bars represent small insertions and deletions

Of the 8667 variants, 174 were shared by all the cases, which were listed in Additional file 4. Ninety-six percent of the shared variants were SNVs, and 14.4% and 2.9% were recorded in COSMIC and ClinVar database, respectively. None of the variants was predicted to be deleterious based on combined annotation-dependent depletion (CADD) score ≥ 10. Those variants located in 123 genes, and their impact on gene function were illustrated in Fig. 2. Over half of the variants (51.1%) were missense mutations caused by SNVs, 1.1% were frameshift mutations caused by InDels, and 67.8% were nonsynonymous in total.

### Newly identified variants

**Fig. 3:**
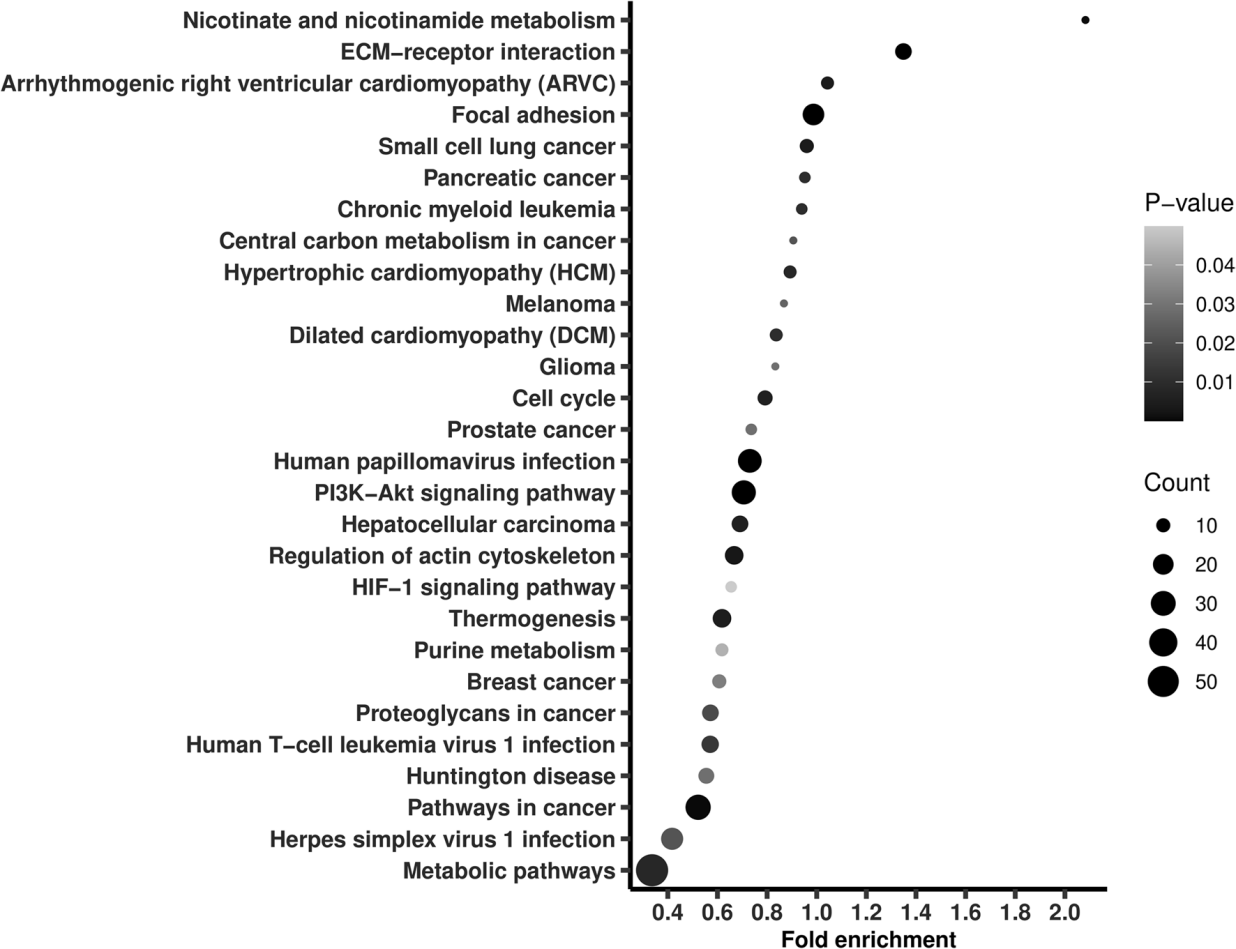
Pathway enrichment of the genes where the newly identified variants located. Fold enrichment is calculated by dividing gene ratio (the number of genes enriched in the pathway divided by the total number of analyzed genes) with background ratio in KEGG database. *P* values were adjusted

We further excluded variants recorded in gnomAD and in dbSNP, identifying 1010 novel variants (see Additional file 5). Ninety percent of the novel variants were SNVs, and only 2% were recorded in COSMIC database and none of them was found in ClinVar database. The novel variants located in 909 genes, on which we also conducted a pathway enrichment. As shown in Fig. 3, the genes were enriched into 28 pathways, including metabolic pathways, pathways in cancer, PI3K-AKT signaling pathway, and chronic myeloid leukemia.

None of the 1010 novel variants was shared by the 10 cases. However, one SNV located in *SESN3* gene was found to be shared by 90% cases. It was recorded neither in COSMIC nor in ClinVar database. The SNV (C > G) located in *SESN3* (chr11:95230844) was a missense mutation (resulting in Gly > Ala).

## Discussion

Despite presenting different clinical types, BIL has been proved to be caused by and is diagnosed primarily on benzene exposure. Recurrent genetic variants may exist among different BIL patients. In order to find variants that may help understand carcinogenic mechanisms of BIL and finally make more effective prevention and treatment measures, we carried out the WES on peripheral blood samples of 10 BIL cases, and identified 48,802 variants in exons in total, 97.3% of which were SNVs. After filtering out variants with MAF ≥ 1%, we obtained 8667 potentially pathogenic variants, of which 174 were shared by all the BIL cases. We also identified 1010 novel variants that might be specifically related to BIL, and one of them was shared by 90% cases.

Because paired normal samples of the cases were not available, we could not accurately differentiate somatic variants from those of germline. By using MAF < 1% as the cutoff value, which is recommended by the Interpretation of Sequence Variants in Somatic Conditions Working Group and also commonly used across many clinical laboratories [26], we obtained variants that were very likely to be somatic and disease-related. However, the number of the variants was much larger than averagely 13 per sample in de novo AML or 8 per sample in newly diagnosed CML as previously reported by the Cancer Genome Atlas Research Network and Togasaki et al. [27, 28] Apart from naturally acquired and accumulated through the long-term course of BIL, some variants might be introduced by the therapies. Unfortunately, we could not exclude such variants in this study due to the fact that BIL is usually diagnosed as occupational cancer long after it is clinically diagnosed and treated in China.

The identified variants located in a wide series of genes, some of which have been reported before, such as *TP53*, *TET2*, *SF3B1* and *PTPN11* in AML [27, 29], and *ASXL1*, *TET2*, *TET3*, *CENPF*, *TLE1*, *PRDM9*, *TTN*, *COL7A1* and *DLK1* in CML [13, 14, 28]. Those mutated genes have been suggested to play important roles in carcinogenesis. The following pathway analysis showed that some of the mutated genes were significantly enriched into certain popular cancer-related pathways, such as PI3K-AKT signaling pathway and Ras signaling pathway. The PI3K-AKT-mTOR pathway was constantly found activated in a variety of cancers. The PI3K-AKT-mTOR axis consists of many regulators of oncogenic potentials, including the catalytic (p110α) and regulatory (p85α), of AKT, class IA PI3K, mTOR, RHEB, and eIF4E [30]; it may prompt oncogenic transformation by the mechanisms including stimulation of proliferation, metabolic reprogramming, invasion/metastasis, survival, and suppression of autophagy and senescence [30]. The Ras signaling pathway is one of the main pathways to transduce intracellular signals in response to mitogens to control cell growth, survival and anti-apoptotic programs. Three *RAS* genes encode four main protein products: KRAS4A, KRAS4B, NRAS and HRAS [31]. RAS proteins cycle between the GDP-bound inactive state (RAS-GDP) and the GTP-bound active state (RAS-GTP) [31]. The active RAS-GTP interacts with downstream effector enzymes including RAF, PI3K, and Ral guanine exchange factors (RalGEFs), transducing the signal to regulate biological behavior [32–35]. Ras pathway mutations occur in approximately 19% of all cancers, playing a prominent role in tumorigenesis and tumor progression [36].

We further examined recurrence of the variants and found that some variants were identified in each of the BIL cases, which gives *P* values no more than 8.8 × 10^−14^. The result suggested that the shared variants were significantly related to BIL. As previously inferred, some of the shared variants might be involved in the process of carcinogenesis, some might occur through the development of the disease, and others might be induced by the therapies.

By excluding variants recorded in the public databases, we obtained novel variants in BIL. Some of the novel variants might be specifically related to BIL, while others might be previously undiscovered, non-specific variants. Some of the novel variants were also located in genes that can be enriched into cancer-related pathways such as PI3K-AKT signaling pathway, suggesting that they might participate in the carcinogenic mechanisms of BIL.

When examining the recurrence of the novel variants, we found one variant shared by 90% cases. It locates in *SESN3* gene. The *SESN3* gene encoded protein (Sestrin 3) belongs to a small protein family that has been implicated in multiple biological processes including anti-oxidative stress, anti-aging, cell signaling, and metabolic homeostasis [37]. Sestrin 3 was suggested to play a critical tumor suppressor role through multiple mechanisms, including inhibition of the hedgehog signaling, controlling regeneration of peroxiredoxins to balance reactive oxygen species (ROS) upregulation induced by oncogenic Ras [37–39]. In BCR-ABL expressing cells, *SESN3* mediated anti-leukemic responses through inhibition of mTOR signaling cascade [40]. Mutations in this gene might eventually result in oncogenesis.

## Conclusions

To sum up, we examined variations of the whole exome in BIL patients for the first time. Although cases of different clinical types were not analyzed respectively due to limited sample size, we identified among all the cases some commonly shared variants that might be related to BIL. We also found novel variants in the exons of BIL cases, and one of them located in a cancer-related gene was shared by most of the cases, suggesting that it might be specifically related to BIL and has potential impact on biological structures or functions of the gene. Our study provided preliminary, genetic information for unraveling carcinogenic mechanisms of BIL.

## Supplementary Information

**Additional file 1.** Statistics of the whole-exome sequencing data of the benzene-induced leukemia cases.

**Additional file 2.** Statistics of the sequences alignment.

**Additional file 3.** Whole-exome sequencing identified genetic variants with minor allele frequency < 1% in the gnomAD database for East Asian.

**Additional file 4.** Whole-exome sequencing identified genetic variants with minor allele frequency < 1% in the gnomAD database for East Asian and shared by all cases.

**Additional file 5.** Whole-exome sequencing identified genetic variants with no record in the gnomAD database for East Asian and dbSNP.

## Data Availability

The whole-exome sequencing datasets generated and analyzed during the current study are available in the National Center for Biotechnology Information SRA database, [https://www.ncbi.nlm.nih.gov/Traces/study/?acc=PRJNA830611].

## Abbreviations

ALL: Acute lymphocytic leukemia
AML: Acute myeloid leukemia
AP: Accelerated phase
BC: Blast crisis
BIL: Benzene-induced leukemia
BP: Blast phase
CADD: Combined annotation-dependent depletion
CML: Chronic myeloid leukemia
COSMIC: Catalogue of Somatic Mutations in Cancer
CP: Chronic phase
DNMT3A: DNA methyltransferase 3A
GATK: The genome analysis toolkit
IDH: Isocitrate dehydrogenase
InDel: Small insertion and deletion
KEGG: The Kyoto encyclopedia of genes and genomes database
MAF: Minor allele frequency
ROS: Reactive oxygen species
SNV: Single nucleotide variant
SPTCOD: Shenzhen Prevention and Treatment Center for Occupational Diseases
TKI: Tyrosine kinase inhibitor
WES: Whole-exome sequencing
