# Supplementary material for: Whole-exome sequencing identified recurrent and novel variants in benzene-induced leukemia
Source: BMC Med Genomics. 2023 Jan 26;16:13. doi: 10.1186/s12920-023-01442-w (PMC9878782; doi:10.1186/s12920-023-01442-w)
Supplement: Supplementary file 1 — Additional file 1. Statistics of the whole-exome sequencing data of the benzene-induced leukemia cases. [file 12920_2023_1442_MOESM1_ESM.docx]

**Additional file 1** Statistics of the whole-exome sequencing data of the benzene-induced leukemia cases

| Samples | Raw reads | Raw bases (Mb) | Clean reads | Clean bases (Mb) | Clean data rate (%) | Clean read1 Q20 (%) | Clean read2 Q20 (%) | Clean read1 Q30 (%) | Clean read2 Q30 (%) | GC content (%) |
| --- | --- | --- | --- | --- | --- | --- | --- | --- | --- | --- |
| Case_1 | 150698778 | 15069.88 | 150559252 | 15055.48 | 99.90 | 97.99 | 92.91 | 94.66 | 85.75 | 49.29 |
| Case_2 | 135797102 | 13579.71 | 135684818 | 13565.71 | 99.90 | 97.40 | 94.16 | 93.07 | 86.91 | 49.44 |
| Case_3 | 121515440 | 12151.54 | 121411356 | 12140.60 | 99.91 | 97.83 | 92.22 | 94.21 | 84.48 | 48.96 |
| Case_4 | 139386136 | 13938.61 | 139279714 | 13924.58 | 99.90 | 97.22 | 93.78 | 92.53 | 86.06 | 49.42 |
| Case_5 | 130179532 | 13017.95 | 130081066 | 13004.60 | 99.90 | 97.07 | 93.97 | 92.22 | 86.42 | 49.88 |
| Case_6 | 127287116 | 12728.71 | 127180756 | 12717.64 | 99.91 | 97.83 | 92.13 | 94.16 | 84.25 | 49.25 |
| Case_7 | 140090422 | 14009.04 | 139960986 | 13995.63 | 99.90 | 98.04 | 92.66 | 94.80 | 85.28 | 50.28 |
| Case_8 | 133002760 | 13300.28 | 132886846 | 13288.27 | 99.91 | 97.90 | 92.60 | 94.39 | 85.12 | 49.26 |
| Case_9 | 152635366 | 15263.54 | 152459290 | 15245.44 | 99.88 | 98.01 | 97.81 | 94.51 | 93.72 | 51.47 |
| Case_10 | 150576368 | 15057.64 | 150386318 | 15036.44 | 99.86 | 96.13 | 94.41 | 91.09 | 88.11 | 49.10 |
