# Supplementary material for: Whole-exome sequencing identified recurrent and novel variants in benzene-induced leukemia
Source: BMC Med Genomics. 2023 Jan 26;16:13. doi: 10.1186/s12920-023-01442-w (PMC9878782; doi:10.1186/s12920-023-01442-w)
Supplement: Supplementary file 2 — Additional file 2. Statistics of the sequences alignment. [file 12920_2023_1442_MOESM2_ESM.docx]

**Additional file 2** Statistics of the sequences alignment

| Samples | Initial bases on target | Total effective reads | Total effective bases (Mb) | Effective sequences on target (Mb) | Capture specificity (%) | Mapping rate on genome (%) | Duplicate rate on genome (%) | Mismatch rate in target region (%) | Average sequencing depth on target | Fraction of target covered ≥ 1× (%) | Fraction of target covered ≥ 4× (%) | Fraction of target covered ≥ 10× (%) | Fraction of target covered ≥ 20× (%) |
| --- | --- | --- | --- | --- | --- | --- | --- | --- | --- | --- | --- | --- | --- |
| Case_1 | 60457902 | 122360688 | 12131.90 | 6252.09 | 51.53 | 99.98 | 18.73 | 1.32 | 103.41 | 99.35 | 99.18 | 98.76 | 97.56 |
| Case_2 | 60457902 | 121219823 | 12017.60 | 6069.25 | 50.50 | 99.96 | 10.64 | 1.33 | 100.39 | 99.55 | 99.38 | 98.88 | 97.47 |
| Case_3 | 60457902 | 91904122 | 9102.61 | 4383.34 | 48.15 | 99.92 | 24.26 | 1.43 | 72.50 | 99.56 | 99.35 | 98.61 | 95.90 |
| Case_4 | 60457902 | 119910400 | 11879.40 | 5651.17 | 47.57 | 99.96 | 13.89 | 1.40 | 93.47 | 99.35 | 99.17 | 98.68 | 97.25 |
| Case_5 | 60457902 | 110511724 | 10963.00 | 5156.91 | 47.04 | 99.95 | 15.03 | 1.30 | 85.30 | 99.34 | 99.15 | 98.59 | 96.75 |
| Case_6 | 60457902 | 109632998 | 10858.20 | 5139.91 | 47.34 | 99.98 | 13.80 | 1.49 | 85.02 | 99.53 | 99.36 | 98.78 | 96.85 |
| Case_7 | 60457902 | 115048199 | 11404.10 | 6537.75 | 57.33 | 99.98 | 17.80 | 1.34 | 108.14 | 99.33 | 99.16 | 98.74 | 97.61 |
| Case_8 | 60457902 | 115459010 | 11444.50 | 5558.85 | 48.57 | 99.98 | 13.12 | 1.40 | 91.95 | 99.33 | 99.15 | 98.63 | 97.14 |
| Case_9 | 60457902 | 128006554 | 12781.60 | 7142.80 | 55.88 | 99.99 | 16.06 | 0.43 | 118.15 | 99.52 | 99.36 | 98.94 | 97.75 |
| Case_10 | 60457902 | 114072624 | 11306.50 | 5836.04 | 51.62 | 99.62 | 23.89 | 1.25 | 96.53 | 99.34 | 99.16 | 98.70 | 97.40 |
